# Identification of LncRNA Linc00513 Containing Lupus-Associated Genetic Variants as a Novel Regulator of Interferon Signaling Pathway

**DOI:** 10.3389/fimmu.2018.02967

**Published:** 2018-12-18

**Authors:** Zhixin Xue, Chaojie Cui, Zhuojun Liao, Shiwei Xia, Pingjing Zhang, Jialin Qin, Qiang Guo, Sheng Chen, Qiong Fu, Zhihua Yin, Zhizhong Ye, Yuanjia Tang, Nan Shen

**Affiliations:** ^1^Shanghai Institute of Rheumatology, Renji Hospital, School of Medicine, Shanghai Jiaotong University, Shanghai, China; ^2^Shenzhen Futian Hospital for Rheumatic Diseases, Shenzhen, China; ^3^Center for Autoimmune Genomics and Etiology, Cincinnati Children's Hospital Medical Center, Cincinnati, OH, United States; ^4^Department of Pediatrics, University of Cincinnati College of Medicine, Cincinnati, OH, United States; ^5^State Key Laboratory of Oncogenes and Related Genes, Shanghai Cancer Institute, Renji Hospital, School of Medicine, Shanghai Jiaotong University, Shanghai, China

**Keywords:** single-nucleotide polymorphism, long non-coding RNA, systemic lupus erythematosus, interferon, CRISPR-dCas9

## Abstract

Systemic lupus erythematosus (SLE) is a complex autoimmune disease characterized by augmented type I interferon signaling. High-throughput technologies have identified plenty of SLE susceptibility single-nucleotide polymorphisms (SNPs) yet the exact roles of most of them are still unknown. Functional studies are principally focused on SNPs in the coding regions, with limited attention paid to the SNPs in non-coding regions. Long non-coding RNAs (lncRNAs) are important players in shaping the immune response and show relationship to autoimmune diseases. In order to reveal the role of SNPs located near SLE related lncRNAs, we performed a transcriptome profiling of SLE patients and identified linc00513 as a significantly over expressed lncRNA containing functional SLE susceptibility loci in the promoter region. The risk-associated G allele of rs205764 and A allele of rs547311 enhanced linc00513 promoter activity and related to increased expression of linc00513 in SLE. We also identified linc00513 to be a novel positive regulator of type I interferon pathway by promoting the phosphorylation of STAT1 and STAT2. Elevated linc00513 expression positively correlated with IFN score in SLE patients. Linc00513 expression was higher in active disease patients than those inactive ones. In conclusion, our data identify two functional promoter variants of linc00513 that contribute to increased level of linc00513 and confer susceptibility on SLE. The study provides new insights into the genetics of SLE and extends the role of lncRNAs in the pathogenesis of SLE.

## Introduction

Systemic lupus erythematosus (SLE) is a heterogenous autoimmune disease with complex immune phenotype and diverse clinical manifestations (1, 2). Genetic and environmental factors are considered the two most important pathogenic factors, however the exact etiology of SLE remains obscure (3–5). High-throughput technologies used in genome-wide association studies (GWASs) have identified plenty of important SLE susceptibility loci (6–8) yet the exact roles of these SNPs are still largely unknown. It is interesting to note that the vast majority of the identified SNPs are located in non-coding regions, and some of them have been proven to be functional. For instance, our previous study revealed a variant in miR-146a promoter could regulate miR-146a expression and contribute to SLE disease risk (9). However, most of the functional studies are focused on SNPs in the coding regions (10–13), with limited attention paid to the function of SNPs that located in the non-coding regions, markedly in the regions around disease related lncRNAs.

LncRNAs have been proved to be effective regulators of gene expression and important modulator in diverse biological processes (14–16). Deregulation of lncRNAs was demonstrated to have relevance to aberrant immune response and linked to autoimmune diseases such as multiple sclerosis (MS), rheumatoid arthritis (RA) and SLE (17–19). Although SLE is a highly heterogeneous disease, a large part of the patients have the common feature of high expression levels of interferon (IFN)-inducible genes, which is so called IFN signature (20–22). As recently revealed by hitherto the largest transcriptional profiling of SLE patients, about 80% of the SLE patients have the IFN signature (23). Type I IFN is the cytokine that functions in shaping various immune responses (24–26). Many potential mechanisms have been identified to be implicated in exacerbation of SLE disease by IFN (27–30). Hence type I IFN is thought to be one of the most important signaling pathways involved in SLE pathogenesis. Non-coding RNAs like microRNAs miR-146a and miR-155 have been reported to be effective regulators of type I IFN pathway (31, 32). It is conceivable that lncRNAs may probably also play a non-negligible role in regulating IFN pathway and contribute to SLE disease development.

In an effort to reveal the role of SNPs located near SLE related lncRNAs, we made an attempt to dissect the function of SLE susceptible variants by focusing on those near the candidate lncRNA selected based on high-throughput transcriptome analysis. We identified linc00513 from a group of distinctly over expressed lncRNAs as it contained SLE susceptible SNP in the promoter (6). We demonstrated that linc00513 was a novel positive regulator of IFN signaling pathway and was responsible for the amplified IFN signaling in SLE patients. Our data also provided evidence that SNPs rs205764 and rs547311 in the promoter region of linc00513, which modulate its expression, can affect disease susceptibility of SLE.

## Materials and Methods

### Patients and Ethics Statement

139 SLE patients were recruited for the genotyping and mRNA expression studies. All patients that recruited met the 1997 American College of Rheumatology (ACR) criteria for SLE. The study was conducted in accordance with the principles expressed in the Declaration of Helsinki and was approved by the Research Ethics Board of Renji Hospital, School of Medicine, Shanghai Jiaotong University.

### IFN Scores Calculation

The IFN scores were calculated from the expression data for three representative IFN-inducible genes according to a previously described algorithm (33). In this study we selected three typically type I IFN-inducible genes IFI44, Mx1, and OAS1 to calculate (20, 21, 34). The mean IFN score for the SLE patients was 19.37 ± 22.25, and the mean IFN score for the 21 sex- and age-matched healthy controls was 0 ± 2.82.

### DNA Isolation and Genotyping

Genomic DNA was isolated from human whole blood samples using QIAamp DNA blood kit (Qiagen) and quantified using a NanoDrop spectrophotometer (NanoDrop Technologies). SNPs rs205764 and rs547311 were genotyped with specified TaqMan SNP genotyping probes (Applied Biosystems) following the manufacturer's recommendations for allelic discrimination in the QuantStudio™ 7 Flex Real-Time PCR System (Applied Biosystems).

### RNA Sequencing

RNAs isolated from 22 SLE patients and 7 sex- and age-matched controls renal tissues were qualified by agarose electrophoresis and Agilent 2100 bioanalyzer system under a criteria of 260/280 within 1.8- 2.0, RIN >7, 28S/18S >1.5 and concentration >50 ng/μl. Strand-specific cDNA library was generated from 3μg of RNA using NEBNext Ultra Directional RNA Library Prep Kit for Illumina (NEB, New England Biolabs) after removal of rRNA by Ribo-Zero Gold rRNA Removal Kit (Illumina). Libraries were then sequenced on a Illumina HiSeq 4000 instrument with paired-end reads 150 bp per sample. Filtered and trimmed reads were mapped to human genome reference sequence (UCSC hg38), count the mapped reads to estimate transcriptome abundance. Differential expression analysis was performed using R software. The threshold to define up-regulation was fold change >2 and *p* < 0.05.

### Antisense Oligonucleotides (ASOs) and Constructs

ASOs were designed from Sfold website according to a set of principles and synthesized by Sangon Biotech, Shanghai. Seeded hela cells were transfected with 200 nM ASO or negative control using Lipofectamine RNAimax (Invitrogen) according to the manufacturer's instructions for 24 h and then either treated with 1,000 units/ml type I IFN (PBL) according to the experimental needs. To compare the activity of linc00513 promoter containing the two different alleles, linc00513 promoter–luciferase reporter vectors containing four different combinations of alleles were generated by cloning a 1.12 kb region upstream and approximate to the TSS of linc00513 (−684 to +439) into the pGL3-basic luciferase vector (Promega). To create dCas9/CRISPRi lncRNA expression regulation system, single-guide RNAs (sgRNAs) were designed on Optimized CRISPR Design Website (http://crispr.mit.edu), and cloned either into sgRNA vector that constructed from pEMT vector backbone by our laboratory or into SAM-sgRNA vector that kindly provided by Professor Feng Zhang from MIT. DCas9-Krab and dCas9-VP64 vector were kindly provided by Professor Lei S. Qi from Stanford University and Professor Feng Zhang from MIT, respectively. The sequences of the ASOs, sgRNAs and cloning primers of promoter used in this study were listed in the Supplementary Materials (Table S1, Figure S1).

### Cell Culture, Stimulation, and Transfection

Hela and THP-1 cells were obtained from the Cell Bank, Shanghai Institutes for Biological Sciences, Chinese Academy of Sciences and grown in Dulbecco's modified Eagle's medium (Gibco) or RPMI 1640 (Gibco) containing 10% fetal bovine serum (Gibco). PBMCs were isolated freshly from human peripheral blood using Ficoll-Paque (GE Healthcare). Neutrophils were isolated from the buffy coat after lysis of red blood cells. PBMCs and neutrophils were cultured in RPMI 1640 (Gibco) supplemented with 10% FBS. All cells were maintained at 37°C with a 5% CO_2_ atmosphere. Type I IFN (PBL) was added in the final concentration of 1,000 units/ml. Plasmids and ASOs were transfected into hela cells with Lipofectamine 2000 (Invitrogen) and Lipofectamine RNAiMAX (Invitrogen) according to the manufacturer's instructions, respectively. Plasmids, ASOs and transfection reagents were diluted with Opti-MEMI medium (Gibco) and incubation at room temperature for 20 min after gently mixed, and then the transfection mixture was added to the cell culture. Fresh media were exchanged 5 h after transfection.

### RNA Extraction and Real-Time PCR

Total RNA was extracted using TRIzol (Ambion), and then cDNA was synthesized by reverse transcription using PrimeScript RT Reagent kit (Takara) followed by amplification and quantification by real-time PCR with SYBR Premix Ex Taq™ kit (Takara) in QuantStudio™ 7 Flex Real-Time PCR System (Applied Biosystems). The relative expression levels of target genes and lncRNA were calculated using the 2^−ΔCT^ method normalized to GAPDH. The primers used in the experiments were shown in the Supplementary Materials (Table S1).

### Reporter Gene Assay

One hundred nanogram of 1.12 kb–linc00513 promoter luciferase reporter vector or control pGL3-basic luciferase vector together with 10 ng of Renilla vector were transfected into each well of hela cells that seeded in 96-well plate. Twenty four hours after transfection, cell lysates were added to a 96-well black flat bottom microplate (Greiner Bio-one) and their luciferase activities were measured on a CENTRO XS3 LB 960 luminometer (Berthold) using Dual-Luciferase Reporter Assay System (Promega). The ratio of firefly to Renilla luciferase of each well was calculated and analyzed. All experiments were performed in triplicate or quadruplicate.

### Rapid Amplification of cDNA Ends (RACE)

RACE was performed using SMARTer RACE 5′/3′ Kit (Clontech, Takara) according to manufacturer's instructions to identify the whole sequence of linc00513 transcripts. Briefly, total RNA was extracted freshly from hela cells and 3′- and 5′-RACE-ready cDNAs were synthesized using SMARTScribe Reverse Transcriptase. The amplified PCR products were purified by electrophoresis in 1% agarose gel followed by gel extraction. The purified PCR fragments were cloned into linearized pRACE vector and then sequenced. 3′- and 5′-RACE gene-specific primers (GSPs) were designed according to the reads sequence obtained from RNA-seq. The 3′- and 5′- GSPs and nest GSPs sequences were available in the Supplementary Materials (Table S1).

### Fluorescence *in situ* Hybridization

Hela cells were seeded and grew on the surface of a poly-L-lysine prepared slide inside a 10-cm cell culture dish and then fixed with ethanol. Fixed cells were permeated with DEPC treated 0.1% tritonX-100 for 15 min and wished twice with PBS. Slides were successively treated with SSC, 75, 85, and 100% ethanol and then dried at room temperature. Cells on the slides were detected with 100 μl of the pre-heated hybridization buffer containing probe by incubating in the dark at 37°C in a humidified chamber over night. 5 ng/ml DAPI was used to counter stain the nuclei in the dark at 37°C for 30 min. Slides were washed and observed under a fluorescence microscope. Probe used in FISH experiment was 5′CY3 modified. Probe sequence was shown in the Supplementary Materials (Table S1).

### Western Blotting

Hela cells were seeded at 4 × 10^5^/well in a 6-well plate and transfected with ASO at the final concentration of 200 nM for 24 h. Then cells were stimulated with IFN (1,000 units/ml) for 15 min or 1 h and lysed with RIPA buffer (Pierce, Thermo Scientific) supplemented with protease inhibitor cocktail (Pierce, Thermo Scientific). The cell protein was loaded to SDS-PAGE gel electrophoresis and blotted with the appropriate antibodies. Band signals were visualized with a SuperSignal West Pico kit (Pierce, Thermo Scientific). The antibodies used were as follows: GAPDH rabbit mAb (HRP conjugate), STAT1 rabbit mAb, phospho-STAT1 rabbit mAb, STAT2 rabbit mAb, phospho-STAT2 rabbit mAb, IRF9 rabbit mAb, HRP-linked anti-rabbit IgG. All the antibodies were from Cell Signaling Technology. The primary antibodies were diluted by 1:1,000. The secondary antibody was diluted by 1:2,000.

### Flow Imaging Cytometry

Hela cells transfected with NC or ASOs were stimulated with IFN (1,000 units/ml) for 30 min and then fixed and permeated with eBioscience transcription factor staining buffer set (Invitrogen) according to the manufacturer's protocol. Cells were resuspended in 100 μl of FACS buffer and incubated with antibody (diluted by 1:50) for at least 30 min at room temperature in dark. DAPI was used to stain cell nuclei for <3 min. Cells were then washed and resuspended in FACS buffer (volume between 20 and 200 μl) in an appropriate cell concentration of 1–2 × 10^7^/ml. Cell samples were loaded and analyzed using Amnis ImageStream MarkII (Merck). Similarity between STAT1 and nuclei staining pattern were calculated. The antibody used in flow imaging cytometry experiment was STAT1 rabbit mAb (PE Conjugate; Cell Signaling Technology).

### Data Analysis

Statistical tests were performed with GraphPad Prism software, version 5.01. Figure data are expressed as the arithmetic mean ± SEM. The nonparametric Mann–Whitney *U*-test was used to compare linc00513 expression between the genotype groups and gene expression between the patients and controls. The unpaired *t*-test was used to compare the expression of genes and the luciferase activity of the reporter genes. Spearman's test was used for correlation analysis. Two-tailed *p*-values < 0.05 were considered to be statistically significant. Linkage disequilibrium (LD) patterns were analyzed and displayed with HaploView (35).

## Results

### Linc00513 Is an Over Expressed LncRNA in SLE Containing Disease Susceptibility Loci in the Promoter Region

**Figure 1:**
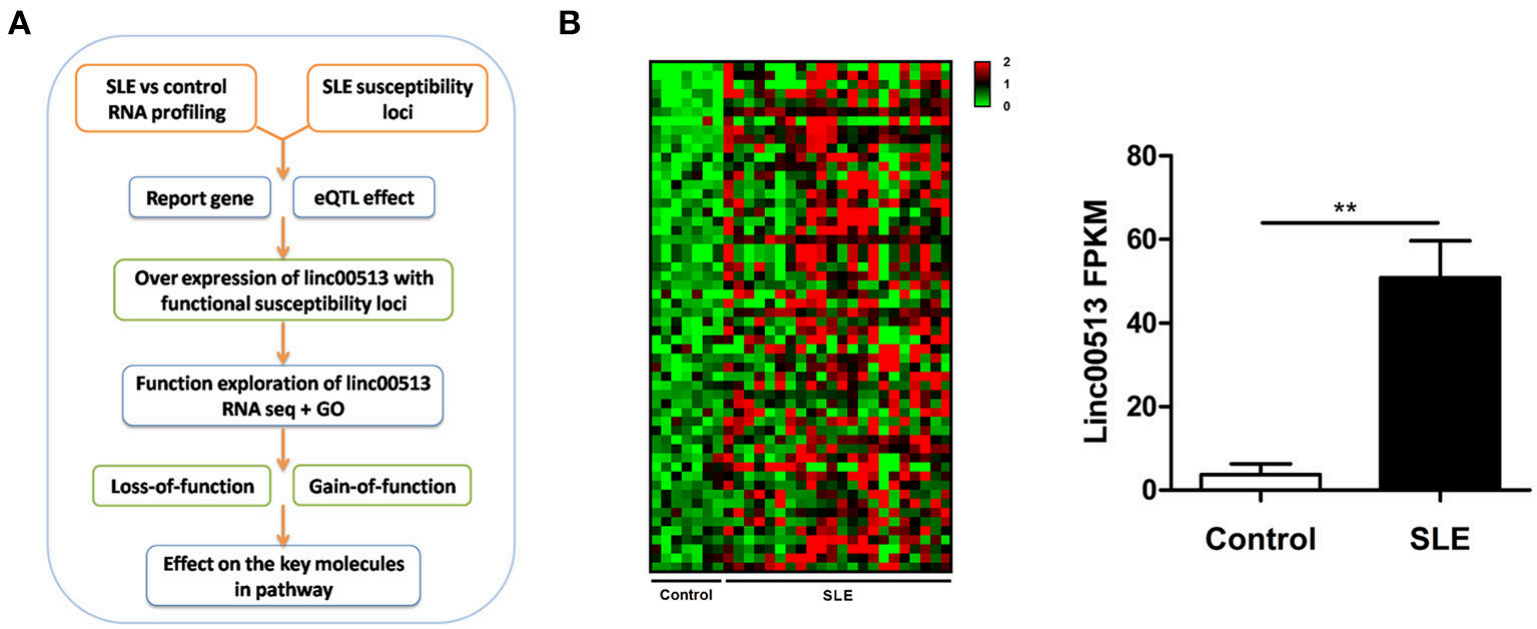
Identification of linc00513 as a significantly over expressed lncRNA in SLE that contains lupus susceptibility SNP in the promoter region. **(A)** Schematic diagram of the whole experiment process. **(B)** 56 lncRNAs showed significant over expression in transcriptome profiling of renal tissues from 22 SLE and 7 controls. FPKM of linc00513 in SLE and control samples were compared using non-parametric Mann-Whitney *U*-test, ^**^indicates *p* < 0.01. Linc00513 ranked the second over expression and meanwhile contained lupus susceptibility SNPs approximate to the transcriptional starting region mentioned by previous GWAS report.

**Figure 2:**
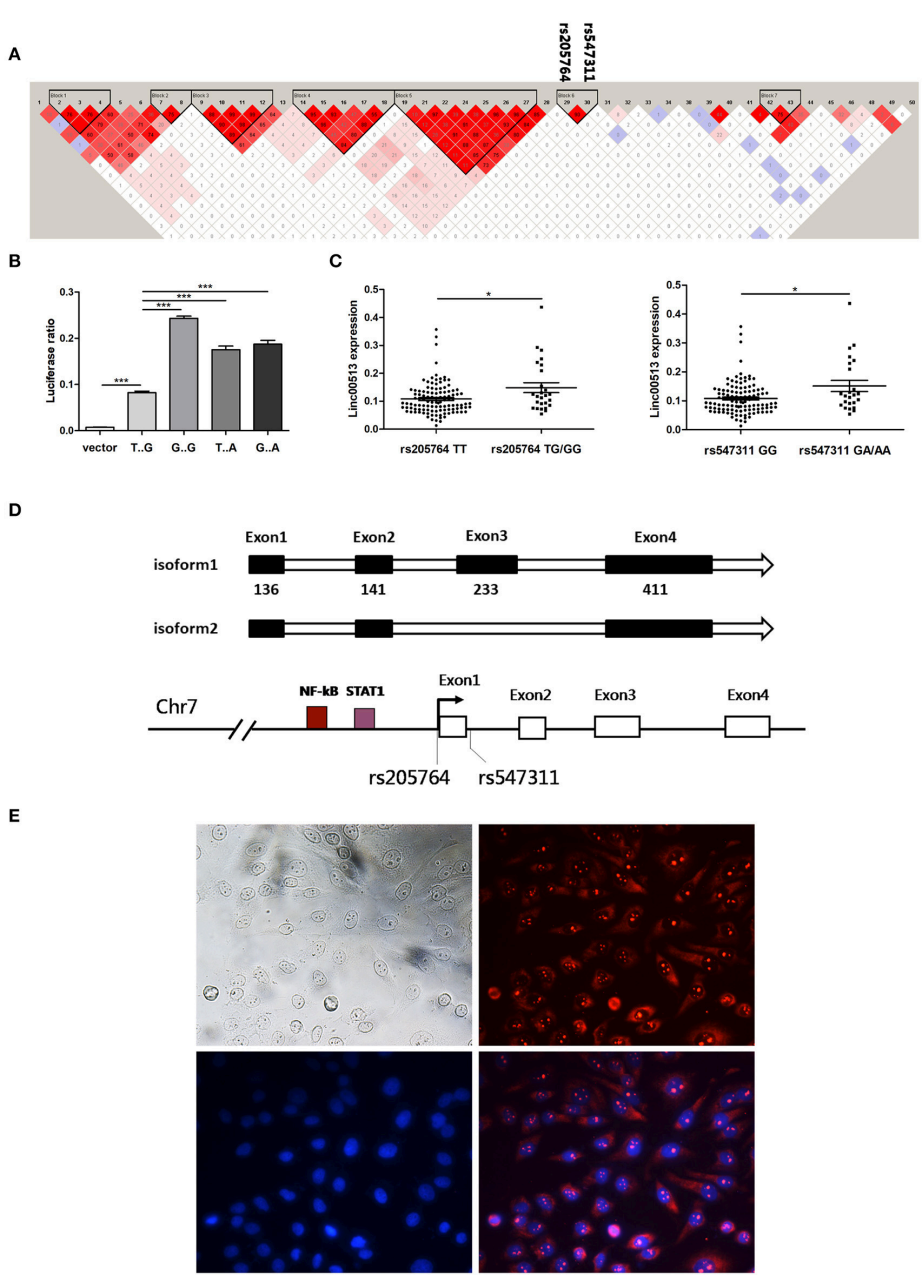
Rs205764 and rs547311 could modulate the expression of linc00513 expression. **(A)** LD patterns of 50 human disease related SNPs in the gene region. Two haplotype blocks were constructed based on the strength of LD in each gene region. **(B)** Luciferase reporter gene assay of linc00513 promoter. 1.12 kb approximate to the TSS of linc00513 (−684 to +439) was cloned into the pGL3-basic luciferase vector. HeLa cells were transfected with linc00513 promoter or control pGL3-basic luciferase reporter vector and Renilla reporter vector. 24 h after transfection, cells were lysed and ratio of firefly to Renilla luciferase activity was analyzed. The data shown are means ± SEM and are representative of three independent experiments performed in triplicate or quadruplicate. *P*-values were analyzed with two-tailed unpaired *t*-test. ***indicates *p* < 0.001. Rs205764 G allele and rs547311 A allele improved linc00513 promoter activity. **(C)** Relative expression level of linc00513 was measured for the different genotypes of rs205764 and rs547311 in neutrophils from 139 SLE individuals (rs205764: TT, *n* = 113; TG, *n* = 24; GG, *n* = 2. rs547311: GG, *n* = 115; GA, *n* = 22; AA, *n* = 2.) *P*-values were analyzed with non-parametric Mann-Whitney *U*-test. *indicates *p* < 0.05. **(D)** Gene structural models of linc00513 as determined by 3′ and 5′ RACE. **(E)** Nuclear and plasma distribution of linc00513 in hela cells as showed by FISH. Upper left: white light; upper right: linc00513 (red); lower left: DAPI (blue); lower right: merge of linc00513 and DAPI.

To identify the aberrant LncRNA expression profile in SLE, we performed transcriptome analysis in 22 lupus patients and 7 controls renal tissues and found 56 significantly over expressed lncRNAs with fold change >2 and *p*-value < 0.05 (Table S2). Then we selected candidate lncRNA from the list using two approaches. The first approach took advantage of differentially expressed lncRNAs profiling in SLE. LncRNAs that aberrantly expressed as compared with controls were selected. In the second approach, we focused on the SNPs identified by previous reported GWAS to have association with lupus susceptibility that closely located to the candidate lncRNAs (Figure 1A). Linc00513 was then chosen for further analysis as it ranked the second over expression and meanwhile contained lupus susceptibility SNP rs547311(G>A) approximate to the transcriptional starting region (Figure 1B). According to the GWAS of SLE in a Chinese Han population genotyping 1,047 cases and 1,205 controls, the minor A allele (14.95%) of rs547311 confer risk on SLE, odds ratio [OR] = 1.46, *p* value = 3.88 × 10^−4^ (6). We also analyzed another SNP rs205764 (T>G) in close proximity to linc00513 transcriptional starting site in the HapMap database. The two SNPs had very high LD (*r*^2^ = 0.9. Figure 2A), which implied the two SNPs in linc00513 promoter region, rs205764 along with rs547311 could play a role in the pathogenesis of SLE.

### Rs205764 and Rs547311 Modulate Linc00513 Expression

According to GWASdb database (36, 37), up to 50 SNPs in 400 kb region around rs205764 and rs547311 have been reported to show human disease susceptibility, which indicates the gene region could be important to human diseases. In order to examine whether the two SNPs in the promoter region of linc00513 were functional, we cloned the promoter of linc00513 (1.12 kb, from −684 to +439) carrying different SNP alleles into the pGL3-basic dual fluorescent reporter gene vector, and determined that the minor alleles (G of rs205764 and A of rs547311) significantly enhanced the transcriptional activity of the linc00513 promoter (Figure 2B). We further tested whether different alleles were associated with linc00513 expression level in SLE patients. Because many confounding factors could disturb the result of expression quantitative trait loci (eQTL) effect, it is preferable to study gene expression in a single cell subset. We examined the eQTL effect in neutrophils because of their good availability and good representativeness of peripheral blood cells. Meanwhile, neutrophils were recently considered very important player in lupus pathogenesis (23, 38–40). We genotyped 139 SLE patients and quantified linc00513 expression in their neutrophils. Patients with TG/GG of rs205764 or GA/AA of rs547311 showed higher expression level of linc00513. So rs205764 (T>G) and rs547311 (G>A) presented eQTL effects on linc00513 in SLE patients (Figure 2C).

Since we have demonstrated that linc00513 was distinctly high expressed in SLE and its promoter could be propelled by two SLE risk related SNPs, so linc00513 could probably be an important lncRNA in SLE. We identified the whole sequence of linc00513 transcript in hela cells using RACE technology, and determined a 921 nt 4-exons isoform of linc00513. An alternative isoform lacking the third exon was also found (Figure 2D, Figures S2, S3). On the basis of whole sequence identification, specific probe was designed for FISH experiment to explore its subcellular localization. As the result revealed, linc00513 showed punctate aggregation distribution in the nucleus with partially dispersed in the cytoplasm (Figure 2E).

### Identification of Linc00513-Regulated Genes

**Figure 3:**
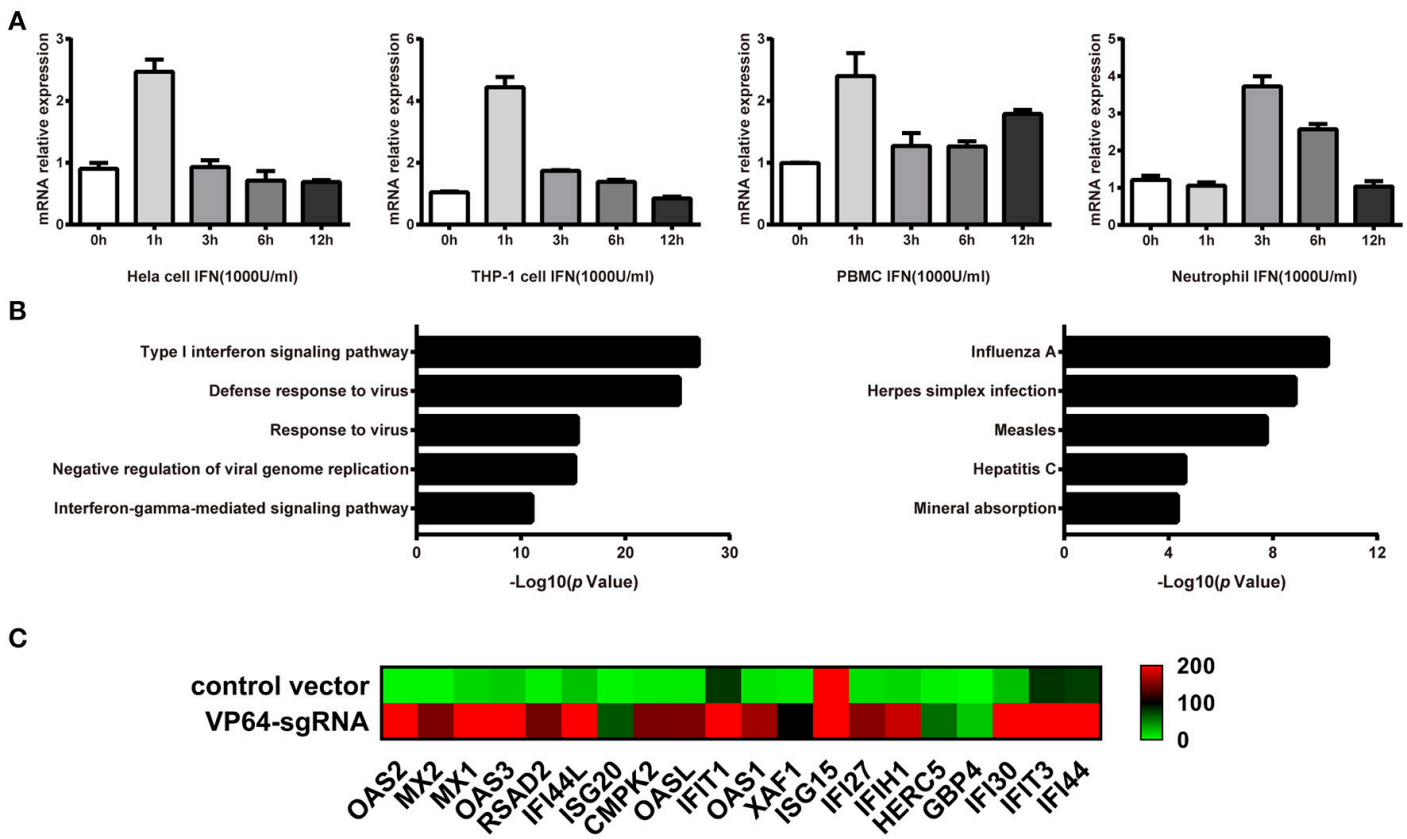
Identification of genes that are regulated by IFN-stimulated lncRNA linc00513. **(A)** Induction of linc00513 by 1,000 U/ml type I IFN in four different cell types on indicated time points, 0, 1, 3, 6, 12h. **(B)** GO and Pathway analysis of linc00513-regulated genes from RNA-seq result in hela cells. **(C)** Expression change of ISGs in hela cells after up-regulation of linc00513 by CRISPR/dCas9-VP64 as compared with cells transfected with empty vectors.

Genomatix prediction revealed the possible binding sites of STAT1 and NF-kB in linc00513 promoter, which were crucial transcription factors downstream of IFN signaling pathway. This result suggested linc00513 might be an IFN-stimulated lncRNA. In order to verify this issue, we treated hela cells with 1,000 U/ml type I IFN and found linc00513 could be induced by IFN with strongest induction on 1 h (Figure 3A). We also showed the induction of linc00513 in response to type I IFN treatment is not restricted to specific cell types. In addition to hela cells, the induction were also observed in other human cell lines and primary blood cells, including THP-1 cells, peripheral blood mononuclear cells (PBMCs) and neutrophils (Figure 3A). We then intended to detect the exact role of linc00513 in SLE. We overviewed the landscape of genes that might be regulated by linc00513. As CRISPR/dCas9-VP64 vector system could be applied to up-regulate gene transcription *in situ* (41, 42), we constructed CRISPR/dCas9-VP64 vector system by designing a sgRNA targeting linc00513 promoter region to effectively promote linc00513 transcription. Then we performed RNA-seq transcriptome analysis in hela cells and revealed 615 genes significantly changed after up-regulating linc00513 (Fold change >2). Intriguingly, we could see genes regulated by linc00513 were mainly interferon-inducible genes, and GO enrichment and KEGG pathways analysis revealed significant involvement of IFN signaling pathway in linc00513-regulated gene network (Figures 3B,C). These results strongly imply that linc00513 may play an important role in regulating the downstream pathway of IFN.

### Linc00513 Promotes the Expression of Downstream Genes of IFN Pathway

**Figure 4:**
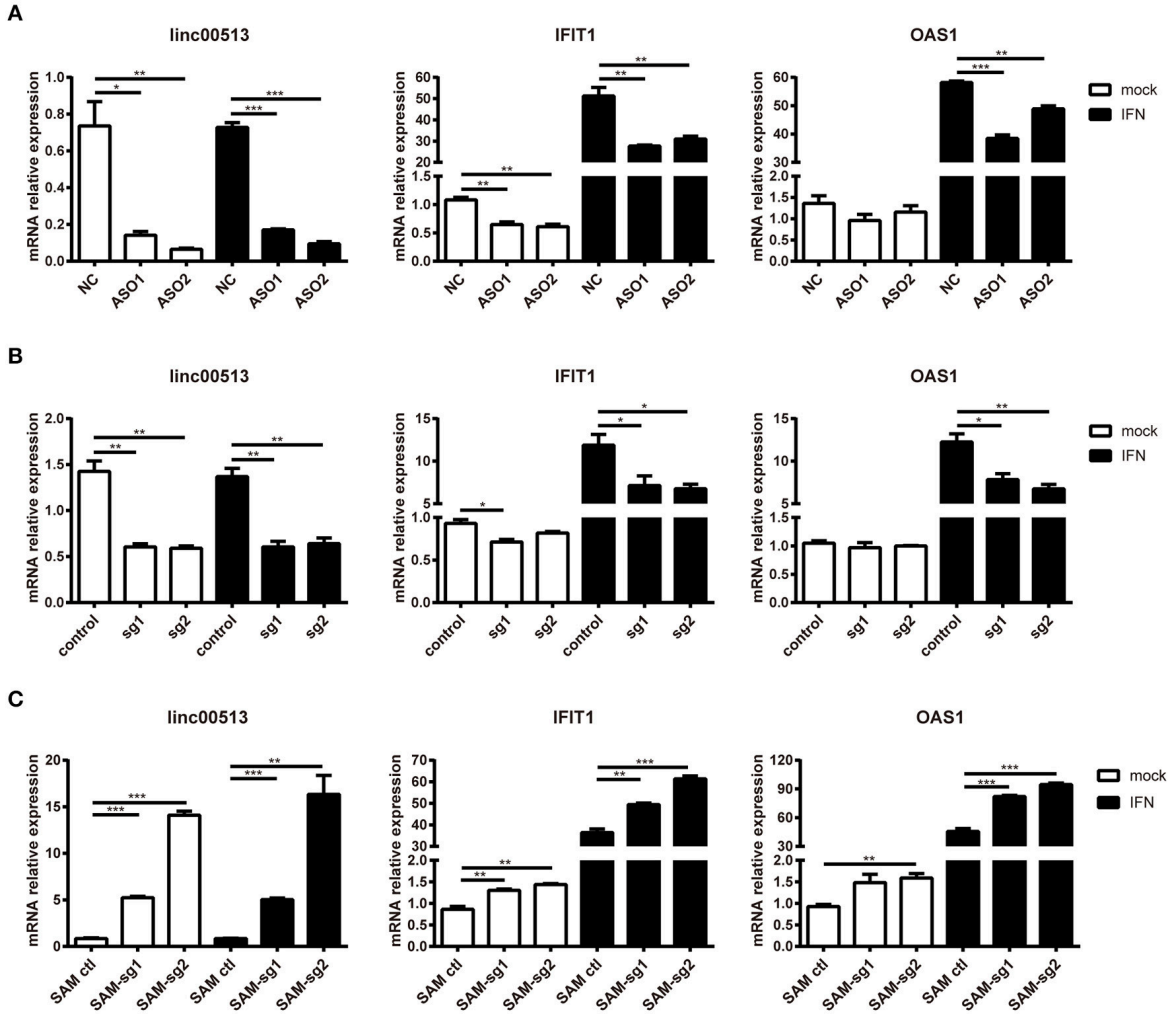
Promotion of the downstream genes of IFN pathway by linc00513. **(A)** Hela cells were transfected with NC or specific ASOs (200 nM) for 24 h and then stimulated with type I IFN. **(B)** Hela cells were transfected with control vectors or specific sgRNA and dCas9-Krab vectors for 24 h and then stimulated with type I IFN. **(C)** Hela cells were transfected with control vectors or specific SAM-sgRNA and dCas9-VP64 vectors for 48 h and then stimulated with type I IFN. Type I IFN was used at the final concentration of 1,000 U/ml for 6 h. The relative expression of linc00513, IFIT1, and OAS1 were analyzed by qPCR. The data shown are means ± SEM and are representative of three independent experiments performed in triplicate. *P*-values were analyzed with two-tailed unpaired *t*-test. *indicates *p* < 0.05, **p < 0.01, ***p < 0.001.

To explore the effect of linc00513 on the IFN signaling pathway, we knocked down the expression of linc00513 in hela cells by two different means, ASOs transfection and CRISPR/dCas9-KRAB vector system. The knock down efficiency of ASOs and CRISPR/dCas9-KRAB vector system were about 75 and 60%, respectively. After knockdown of linc00513, IFN-stimulated gene (ISG) IFIT1 expression was significantly inhibited either with or without IFN stimulation. OAS1 expression also significantly decreased after IFN stimulation, though it didn't significantly decrease without IFN stimulation (Figures 4A,B). We also performed linc00513 up-regulation experiment using CRISPR/dCas9-VP64 vector system. Similarly, up-regulation of linc00513 significantly promoted IFIT1 and OAS1 expression (Figure 4C). Taken together, these data indicate that linc00513 is the positive regulator of the IFN signaling pathway.

### Effects of Linc00513 on the Phosphorylation of Key Molecules in Type I IFN Signaling Pathway

**Figure 5:**
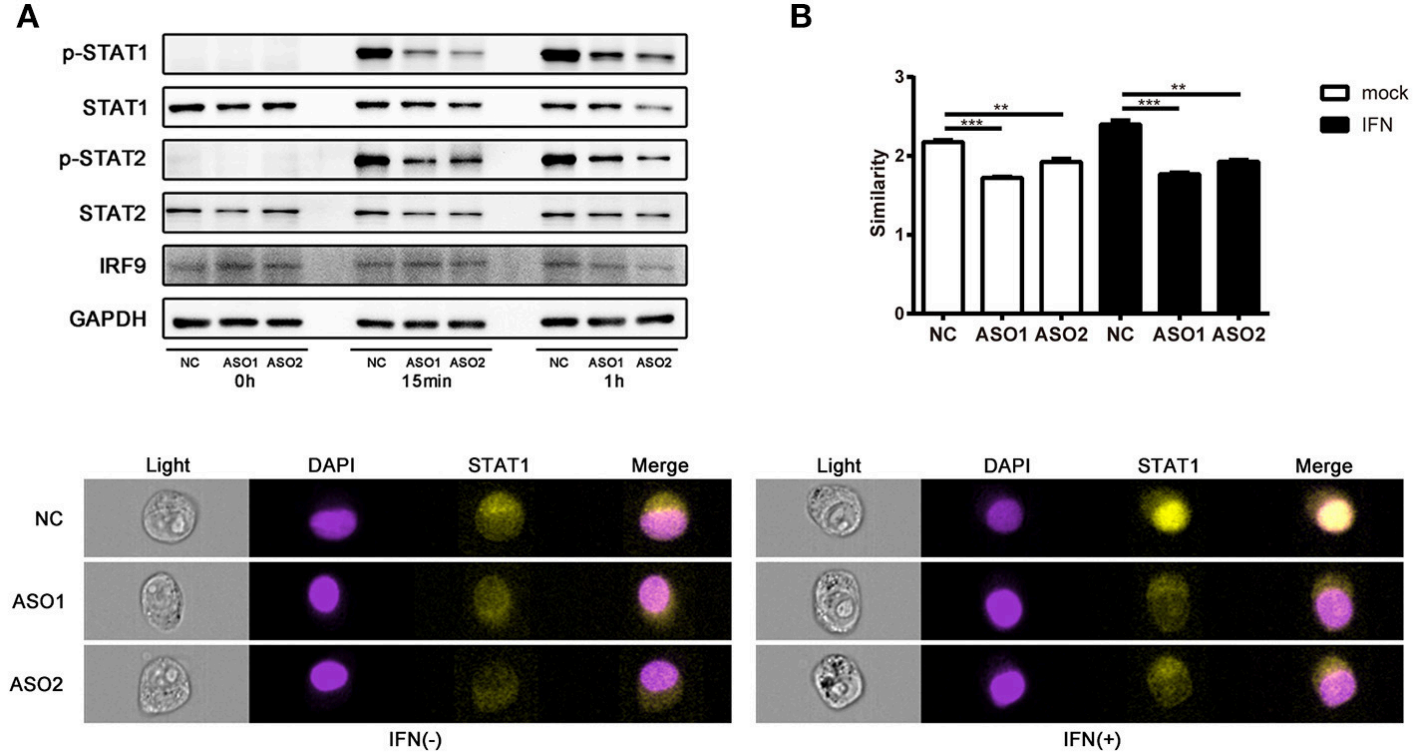
Linc00513 affects the phosphorylation of STAT1 and STAT2 in type I IFN pathway. **(A)** Western blotting of hela cells transfected with NC or specific ASOs (200 nM) for 24 h and then stimulated with type I IFN (1,000 U/ml) for 15 min or 1 h. Blots are representative of three repeated experiments. Quantification of band intensities analysis was shown in Figure S4. **(B)** Flow imaging cytometry of hela cells transfected with NC or specific ASOs (200 nM) for 24 h and then stimulated with type I IFN (1,000 U/ml) for 30 min. Cells were stained with antibody to STAT1 and DAPI after fixation and permeabilization. Values of similarity represent the extent of STAT1 translocation to the nuclei. The data shown are means ± SEM and are representative of three repeated experiments. *P*-values were analyzed with two-tailed unpaired *t*-test. **indicates *p* < 0.01, ***p < 0.001. Cell images are representative of three repeated experiments.

Several lncRNAs that involved in immune pathways have been reported may act through altering the phosphorylation of important transcription factors (43, 44). We tested whether linc00513 played a role in the IFN signaling pathway by modulating key pathway molecules phosphorylation. Western blotting was performed in hela cells tranfected with ASOs targeting linc00513. Knockdown of linc00513 significantly reduced the phosphorylation of STAT1 and STAT2. Intriguingly, to a certain extent, STAT1, STAT2, and IRF9 are also interferon-inducible genes, so it could be explicable that total STAT1, total STAT2, and IRF9 expression also slightly decreased after linc00513 down-regulation (Figure 5A). We then also performed western blotting in hela cells that over expressed linc00513 by CRISPR/dCas9-VP64 vector system transfection. Up-regulation of linc00513 could significantly increase the phosphorylation of STAT1 and STAT2 (Figure S5). Both results supported the role of linc00513 in type I IFN signaling pathway. In type I IFN pathway, the phosphorylation of STAT1 and STAT2 occurs downstream of IFNAR activation, leading to the assembly of the ISGF3 complex which is composed of STAT1-STAT2 dimers and IRF9 (45, 46). ISGF3 translocates into the nucleus and binds to IFN-stimulated response elements (ISRE) in the promoters of IFN-inducible genes to regulate their expression. We further verified the previous result by using flow imaging cytometry and determined the nuclear translocation of STAT1 was reduced after knockdown of linc00513 (Figure 5B). Therefore, we concluded that linc00513 promotes IFN pathway by modulating the phosphorylation of key transcription factors STAT1 and STAT2.

### Association Between Elevated Linc00513 and SLE Disease

**Figure 6:**
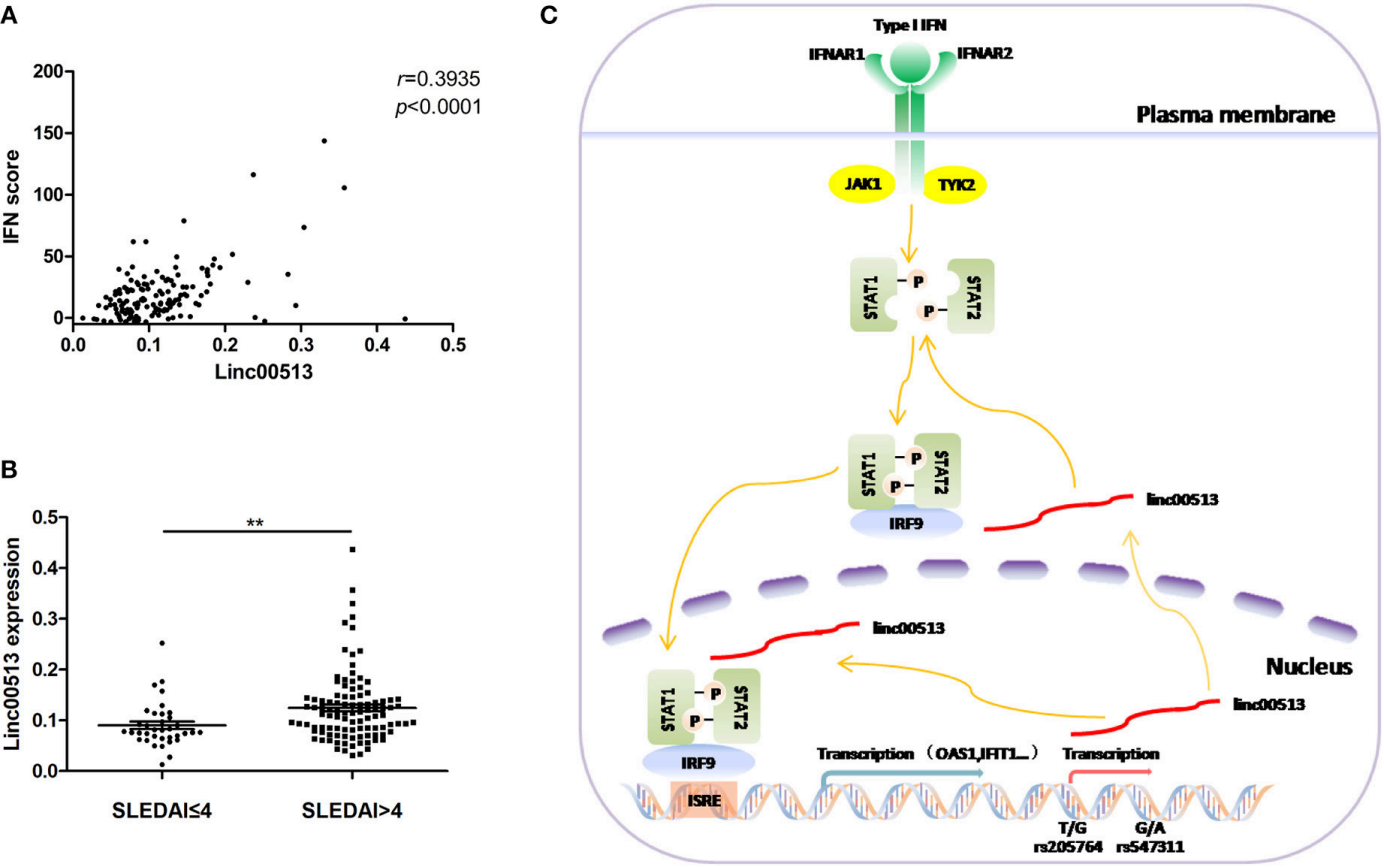
Association between linc00513 and SLE Disease. **(A)** Positive correlation between the expression of linc00513 and IFN scores in SLE patients. *P*-value was determined by Spearman's correlation test. **(B)** The expression of linc00513 was higher in active SLE patients (*n* = 36) than those inactive ones (*n* = 103). *P*-value was determined by non-parametric Mann-Whitney *U*-test, **indicates *p* < 0.01. **(C)** Overview of the modulation of linc00513 by SLE-associated genetic variants and the regulation of type I IFN signaling pathway by linc00513.

Our data have showed the expression of linc00513 was elevated in lupus patients and linc00513 was a positive regulator of IFN pathway. Then we explored whether there was a correlation between linc00513 expression and IFN score in lupus patients. We analyzed neutrophils from 139 SLE patients and found a significant positive correlation between linc00513 expression and IFN score (*r* = 0.3935, *p* < 0.0001; Figure 6A). Clinical information of the patients was listed in Table 1. In addition, we also analyzed the relationship between linc00513 expression levels and SLE disease activity in the same group of patients. Linc00513 expression was higher in active disease patients (SLEDAI-2K > 4) than those inactive ones (SLEDAI-2K ≤ 4; *p* = 0.0017; Figure 6B). Taken together, these data indicate that linc00513 is responsible for the amplified IFN signaling in SLE patients and can contribute to SLE disease activity.

**Table 1:** Demographic, clinical, and laboratory features of the SLE patients.

**Characteristics**	***n* = 139**
Females	130 (93.5%)
Age (years)	33.8 ± 13.2
ANA (+)	139 (100%)
Anti-dsDNA (+)	106 (76.3%)
Anti-Sm (+)	30 (21.6%)
SLEDAI-2K	10.2 ± 5.8

## Discussion

LncRNAs are emerging as indispensable regulators in various biological processes. Aberrations in the lncRNA-mediated immune responses regulation has been linked to autoimmune and autoinflammatory diseases (18, 47, 48). In lupus, over expression of lncRNA NEAT1 was reported to promote secretion of multiple pro-inflammatory cytokines and positively correlated with lupus disease activity (17). The expression of another lncRNA linc0949 was significantly decreased in lupus patients PBMCs and was associated with complement component C3 level and incidence of lupus nephritis (49). While certain lncRNAs have been reported to be involved in SLE pathogenesis, systemic profiling of differentially expressed lncRNAs in SLE is still limited. Our transcriptional profiling in renal tissues of SLE patients and controls revealed abnormally expressed lncRNAs in SLE and identified linc00513 as one of the most significantly over expressed lncRNAs with lupus susceptible SNP loci in the promoter region.

SLE is a complex autoimmune disease with obscure etiology. The type I IFN signaling pathway is recognized to play a pivotal role in SLE pathogenesis among the diverse immunological aberrations present in SLE patients. Several coding genes have been previously identified capable of balancing IFN signaling, like cyclin-dependent kinase 1 (CDK1), a cell cycle regulatory protein gene, could contribute to the over activation of IFN pathway in SLE (50). Our recent research characterized the role of a lncRNA as a positive regulator of the type I IFN signaling by modulating the phosphorylation of key transcription factors STAT1 and STAT2 in this pathway. Knockdown of linc00513 in hela cells reduced the expression of IFIT1 and OAS1, two representative IFN-inducible genes. Similarly, up-regulation of linc00513 promoted ISGs expression. The expression level of linc00513 positively correlated with the IFN score of lupus patients. Thus, we identified linc00513 as a novel robust regulator of type I IFN pathway, providing new evidence for the contribution of non-coding RNAs to the pathogenesis of lupus.

The role of genetic factors in autoimmune disease risk has long been established, however studies on functional exploration of SNPs are quite limited, especially for SNPs in lupus related lncRNA regions. By contrast, it's remarkable that functional studies of cancer related lncRNA SNPs are making continuous progresses. Several specialized databases have even been set up (51, 52). As for lupus, in 2006, six SNPs in the promoter region of Growth arrest-specific 5 (GAS5) had been identified to cause 11-fold down-regulation of the lncRNA expression and correlated with nephritis susceptibility in spontaneous lupus nephritis mouse model BXSB strain (53). Here in our study, we demonstrated rs205764 and rs547311 in the promoter of linc00513 could augment its transcription as determined by reporter gene assay and eQTL effect, thus making linc00513 a distinctly high expressed lncRNA in lupus patients and promoting disease development. To the best of our knowledge, this is the first report to reveal a functional genetic variant in a lncRNA promoter that contributing to SLE disease in human. Our work spotlights the importance of exploring SNP variants in lncRNA regions, which have been more or less ignored in previous genetic studies.

In conclusion, our findings reveal the over expression of linc00513 plays a role in lupus pathogenesis by promoting IFN signaling pathway. SNP variants of the linc00513 promoter are functionally significant in regulating linc00513 expression and conferring susceptibility on SLE (Figure 6C). The study provides new insights into the genetics of SLE and suggests lncRNAs can be novel biomarkers of SLE pathogenesis.

## Author Contributions

All authors were involved in drafting the article or revising it critically for important intellectual content, and all authors approved the final version to be published. ZX, YT, and NS conceived and designed the experiments, ZX, CC, ZL and SX performed the experiments, ZX, CC, and ZL analyzed and interpreted the data, NS, YT, PZ, JQ, QG, SC, QF, ZhY, and ZzY contributed reagents, materials, analysis tools.

### Conflict of Interest Statement

The authors declare that the research was conducted in the absence of any commercial or financial relationships that could be construed as a potential conflict of interest.
