# Supplementary material for: Identification of LncRNA Linc00513 Containing Lupus-Associated Genetic Variants as a Novel Regulator of Interferon Signaling Pathway
Source: Front Immunol. 2018 Dec 18;9:2967. doi: 10.3389/fimmu.2018.02967 (PMC6305415; doi:10.3389/fimmu.2018.02967)
Supplement: Supplementary file 1 [file Data_Sheet_1.pdf]

Supplementary Material

TABLE S1. Sequences of the primers, ASOs and probe used in the study.

**ASOs:**	
ASO1:	GTGTTGGCAGGTGGAGAATG
ASO2:	CTGGCTTAGCGAGGTTGGGT
**sgRNAs:**	
sgRNA1, SAM-sgRNA1 top:	CACCGACTGCAGAAGAATCGCTGTT
sgRNA1, SAM-sgRNA1 bottom:	AAACAACAGCGATTCTTCTGCAGTC
sgRNA2 top:	CACCGGTCACTGCGTGTCATCTCG
sgRNA2 bottom:	AAACCGAGATGACACGCAGTGACC
SAM-sgRNA2 top:	CACCGTAGTTAGCGACCTCTGCTT
SAM-sgRNA2 bottom:	AAACAAGCAGAGGTCGCTAACTAC
**Promoter cloning primers:**	
Forward:	GGTACCAGTTGTCTGTGCGTCCTG
Reverse:	AGATCTCCAGCCTCAGATTGGTTC
**qPCR primers:**	
GAPDH:	
Forward:	GAAGGTGAAGGTCGGAGTC
Reverse:	GAAGATGGTGATGGGATTTC

Linc00513:	
Forward:	ACCAGCATACGGACTCTCAC
Reverse:	GGCGGGAGAATTGCTAAAGG
IFIT1:	
Forward:	GCGCTGGGTATGCGATCTC
Reverse:	CAGCCTGCCTTAGGGGAAG
OAS1:	
Forward:	TCTCAGAAATACCCCAGCCAA
Reverse:	AGAGGACTGAGGAAGACAACCA
IFI44:	
Forward:	GTGCAGGGATGACATATTCTA
Reverse:	ACAAATGCCACACAATGAAT
Mx1:	
Forward:	CAGCACCTGATGGCCTATCA
Reverse:	TGGAGCATGAAGAACTGGATGA
**RACE primers:**	
GSP5:	GATTACGCCAAGCTTGCCTCTCTTCGCATCAGTAGCCCGGACA
Nest-GSP5:	GATTACGCCAAGCTTTCATGCAGGCTGGGCACAGTGGTTC
GSP3:	GATTACGCCAAGCTTGCTGCGGTAGCATTCTCCACCTGCCAA
Nest-GSP3:	GATTACGCCAAGCTTTTCTCCCGCCTTGGCCTCCTAAAGCAC
**FISH probe:**	(5'CY3)-TGTTCTCGCTGATGATGAAACTGGCTT

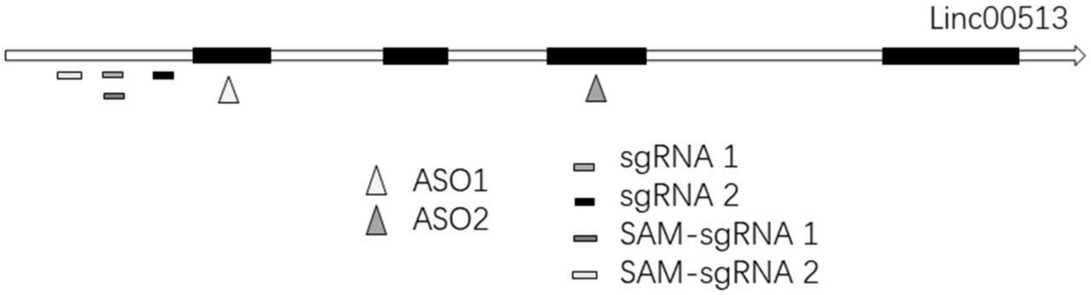

FIGURE S1. Locations of ASOs and sgRNAs.

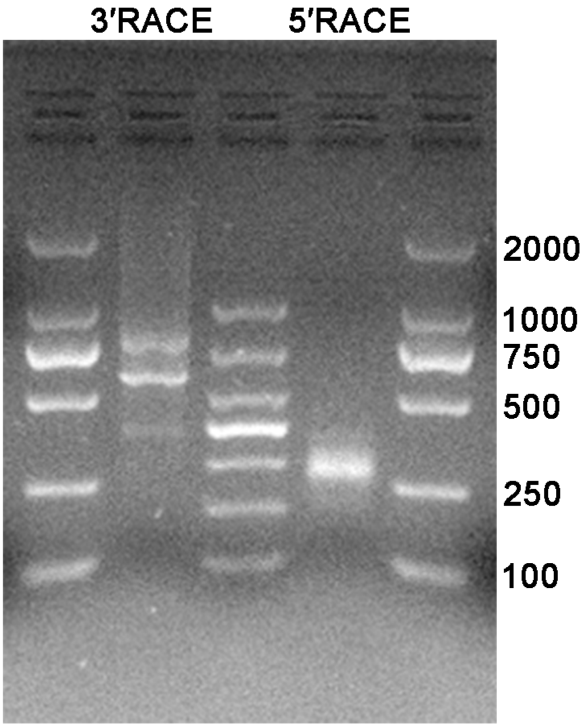

FIGURE S2. Electrophoretic gel bands of 3' and 5' RACE of linc00513 in hela cells

ATGATCCATGCACCCTCTGTCTAATCCTCTGCGATGGGGCCAGTCTGCAGATCCGTCT
GGGCGGGCTGCGGTAGCATTCTCCACCTGCCAACACTCAGTTGCTGGTGAGTAACCA
GCATACGGACTCTCACTTCCTAGATGACGCCTTGCTGTGTTGCCCAGGGTGGTCTCTA
ATTCCTGGCCTTTAGCAATTCTCCCGCCTTGGCCTCCTAAAGCACTGGGGTTACAGGC
ATGAACCACTGTGCCCAGCCTGCATGATGTAATCTCCAAATTGAGGGCAGGAGAGAG
AGTGGTCTCACAACGGAAGGGCACCAGCCACTGAGAAGTCCTAAGGCCGTGTTTGAG
TTCTGGCTCATCTTTTTTGCTAATTTGCTCTTATGCATATCACCCAACCTCGCTAAGCC
AGTTTCATCATCAGCGAGAACAGCACACTGACATGCAGGGCCAGCTCAGGCGCTACC
ACGAGCTACACATCCGTCCATTCGTTTAATCTTCACAGCAGCCCTGGAGGTCACCCAG
GCATGGAATGCACAGCTCTGCCAGACAGAAAGCGCTTTGGATGCCTGAGAAACAAC
AGCTTGTCCGGGCTACTGATGCGAAGAGAGGCCATGGTTTCCTTTTTCTCTGCTCTGA
ATGGCAGCCTTGACGTCACAACAGCTAGGAACCACTGGAGGGACAAATAACAAGGC
GCAAGCCAGAAGCGCCCTGCTCTGTCGCAGGGGCAGGGCCATTTTCATCCAGTTCTG
ATGTCTTCTGTGGCCGCTGCGAGTCGGCGGGAGTAGAGACAAATGACTGGAGCTTTA
GGCGTAAAGGCGGTCTCTCTCTAGGCTGATCAAACAGCCCTTTGCCCAGTGCTAATTG
CCTTCACGGAACCTGGATAAACTACAGTTCCCGAATTAAACTTGTTTCTGATGAACTA
GA

FIGURE S3. Sequence of 4-exon linc00513 in hela cells identified by RACE.

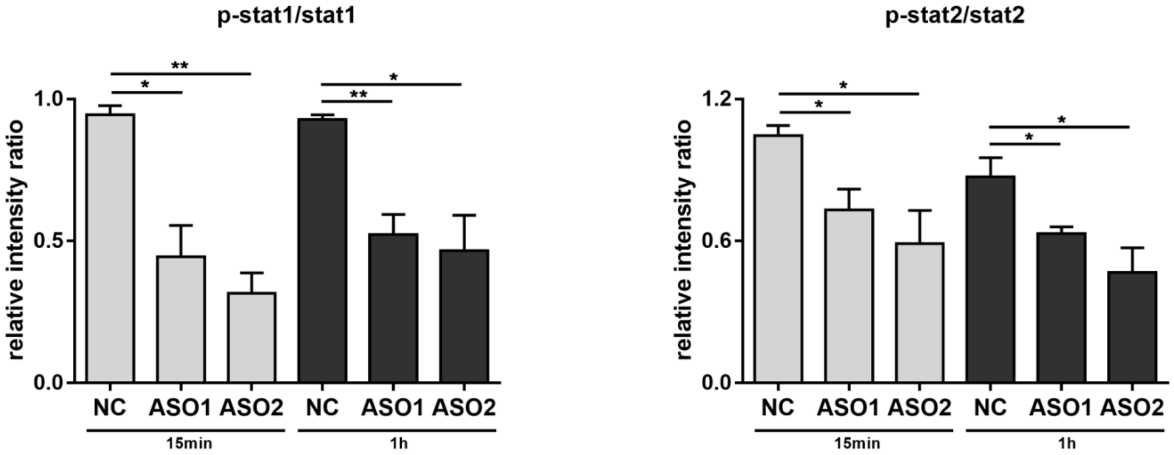

FIGURE S4. Quantification of band intensity analyses. Western blotting band intensity was quantified using Image J and normalized to GAPDH. The ratios of phosphorylated protein to nonphosphorylated protein were calculated, and the ratio at 15 minutes was set as fold 1. * $p < 0.05$, ** $p < 0.01$.

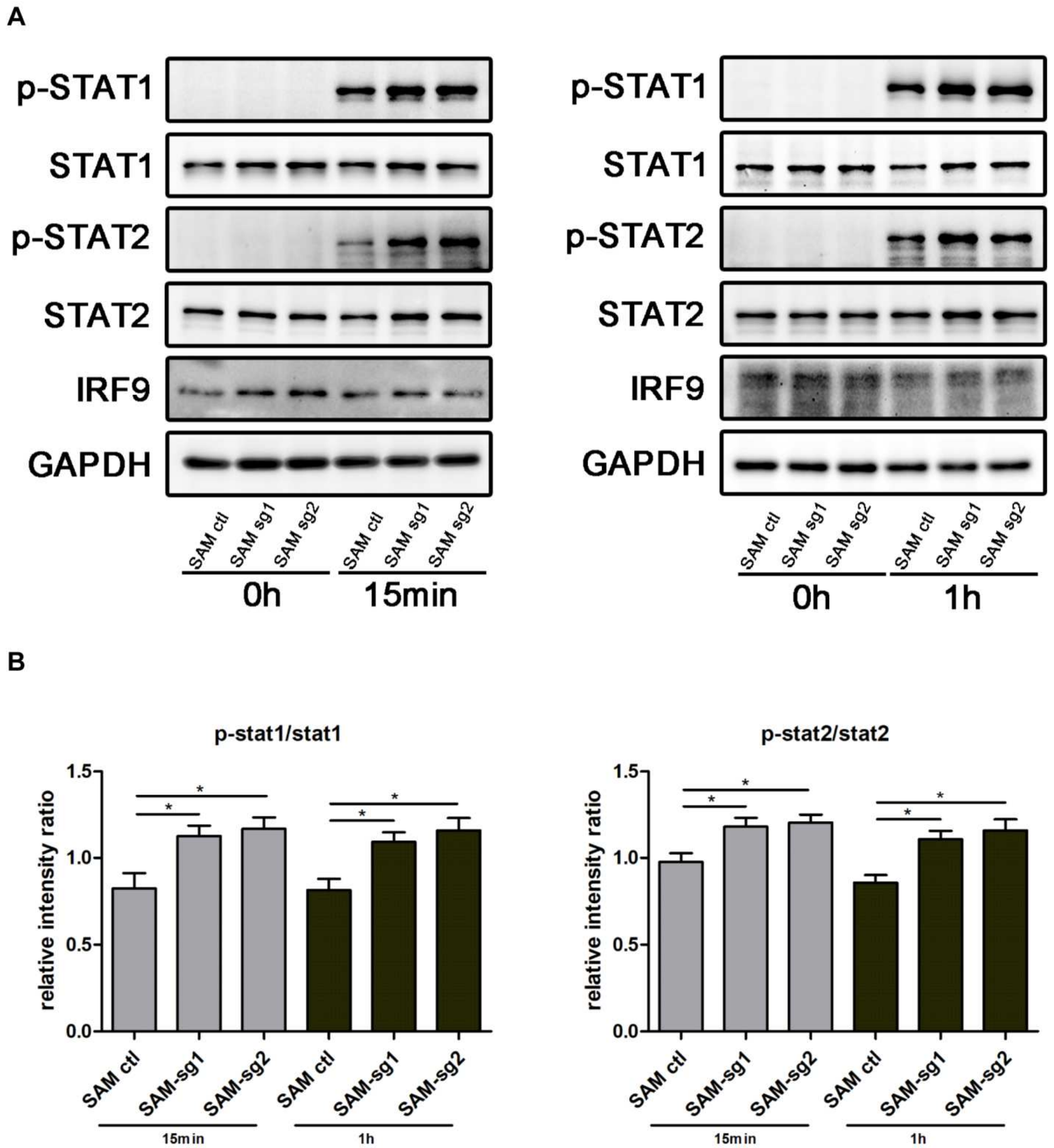

FIGURE S5. Linc00513 affects the phosphorylation of STAT1 and STAT2 in type I IFN pathway. **(A)** Western blotting of hela cells over expressing linc00513. Hela cells were transfected with control vectors or specific SAM-sgRNA and dCas9-VP64 vectors for 48h and then stimulated with type I IFN at the final concentration of 1,000 U/ml for 15 minutes or 1h. The whole-cell lysates were loaded for western blotting. Blots are representative of three repeated experiments. **(B)** Quantification of band intensity analyses. Western blotting band intensity was quantified using Image J and normalized to GAPDH. The ratios of phosphorylated protein to nonphosphorylated protein were calculated, and the ratio at 15 minutes was set as fold 1. The data shown are means ± SEM and are representative of three repeated experiments. *P* values were analyzed with two-tailed unpaired t-test. * indicates $p < 0.05$.
